# Polarity Specific Effects of Transcranial Direct Current Stimulation on Interhemispheric Inhibition

**DOI:** 10.1371/journal.pone.0114244

**Published:** 2014-12-05

**Authors:** Toshiki Tazoe, Takashi Endoh, Taku Kitamura, Toru Ogata

**Affiliations:** 1 Department of Rehabilitation for Movement Functions, Research Institute, National Rehabilitation Center for Persons with Disabilities, Tokorozawa, Japan; 2 Japan Society for the Promotion of Science, Tokyo, Japan; 3 Faculty of Child Development and Education, Uekusa Gakuen University, Chiba, Japan; 4 Division of Functional Control Systems, Graduate School of Engineering and Science, Shibaura Institute of Technology, Saitama, Japan; University of Reading, United Kingdom

## Abstract

Transcranial direct current stimulation (tDCS) has been used as a useful interventional brain stimulation technique to improve unilateral upper-limb motor function in healthy humans, as well as in stroke patients. Although tDCS applications are supposed to modify the interhemispheric balance between the motor cortices, the tDCS after-effects on interhemispheric interactions are still poorly understood. To address this issue, we investigated the tDCS after-effects on interhemispheric inhibition (IHI) between the primary motor cortices (M1) in healthy humans. Three types of tDCS electrode montage were tested on separate days; anodal tDCS over the right M1, cathodal tDCS over the left M1, bilateral tDCS with anode over the right M1 and cathode over the left M1. Single-pulse and paired-pulse transcranial magnetic stimulations were given to the left M1 and right M1 before and after tDCS to assess the bilateral corticospinal excitabilities and mutual direction of IHI. Regardless of the electrode montages, corticospinal excitability was increased on the same side of anodal stimulation and decreased on the same side of cathodal stimulation. However, neither unilateral tDCS changed the corticospinal excitability at the unstimulated side. Unilateral anodal tDCS increased IHI from the facilitated side M1 to the unchanged side M1, but it did not change IHI in the other direction. Unilateral cathodal tDCS suppressed IHI both from the inhibited side M1 to the unchanged side M1 and from the unchanged side M1 to the inhibited side M1. Bilateral tDCS increased IHI from the facilitated side M1 to the inhibited side M1 and attenuated IHI in the opposite direction. Sham-tDCS affected neither corticospinal excitability nor IHI. These findings indicate that tDCS produced polarity-specific after-effects on the interhemispheric interactions between M1 and that those after-effects on interhemispheric interactions were mainly dependent on whether tDCS resulted in the facilitation or inhibition of the M1 sending interhemispheric volleys.

## Introduction

Transcranial direct current stimulation (tDCS) is a widely used interventional brain stimulation technique that improves unilateral upper-limb motor function in healthy humans [1]–[6] and hemiparetic stroke patients [7]–[11]. Based on the polarity-specific after-effects [12], anodal tDCS is applied to the motor cortex innervating the target limb muscles to enhance corticospinal excitability [5], [8], and cathodal tDCS targets the contralateral motor cortex to suppress the contralateral corticospinal excitability [5], [9], which is assumed to contribute to the reduction of transcallosal inhibition from the contralateral side of the primary motor cortex (M1) to the target M1 side [13], [14]. Based on these strategies, recently, anodal and cathodal tDCS are simultaneously applied to one motor cortex and the other, respectively (bilateral tDCS) [3], [5], [6], [10], [15]–[20]. Bilateral tDCS is supposed to combine the effects of anodal and cathodal tDCS and result in larger after-effects compared with unilateral tDCS [3], [10]. However, the advantage of bilateral tDCS is still under debate [15]–[17], [19] because it has not been fully elucidated how tDCS affects transcallosal inhibition underlying interhemispheric balance between motor cortices.

Studies using transcranial magnetic stimulation (TMS) have demonstrated that transcallosal inhibition is affected by the modulation of intracortical motor circuits in both M1 that project and receive callosal volleys [21]–[24]. Hence, it is possible that tDCS-induced neuromodulation in the M1 neural circuits affects transcallosal inhibition. Indeed, Lang et al. [25] demonstrated that transcallosal inhibition measured by the duration of ipsilateral silent period (iSP) was increased and decreased by anodal and cathodal tDCS, respectively, that were unilaterally delivered to the motor cortex receiving transcallosal inhibition. However, the robust effects on iSP were not observed after unilateral tDCS given to the motor cortex projecting callosal volleys [25]. These findings may not be in line with the idea that tDCS given to a motor cortex influences the contralateral motor cortex through the modulation of transcallosal pathways. Subsequently, Williams et al. [5] investigated short-interval interhemispheric inhibition (IHI) elicited by paired-pulse TMS and found that IHI was suppressed after the application of bilateral tDCS combined with unimanual motor training. Although the reduction of IHI was accompanied by the decrease of corticospinal excitability in the side of motor cortex projecting callosal volleys, their causal association was not fully elucidated [5].

IHI and iSP are thought to be mediated by different neuronal populations in the transcallosal pathways [26], suggesting the possibility that tDCS does not affect their different neuronal populations in a similar way. Indeed, Gilio et al. [27] demonstrated that 1 Hz repetitive TMS (rTMS) given to the left M1 suppressed IHI from the left M1 to right M1 with minor effects on iSP. Given these physiological backgrounds, we hypothesized that tDCS given at rest would induce polarity-specific after-effects on IHI from the stimulated M1 in which the corticospinal excitability was changed. To examine this hypothesis, we investigated the after-effects of tDCS applied at rest with three different electrode montages (i.e., unilateral anodal, unilateral cathodal, and bilateral). Each montage was intended to elicit either facilitation of right corticospinal excitability, inhibition of left corticospinal excitability, or both. It should be noted that the intended relative change between the left and right corticospinal excitabilities was the same across the three electrode montages, with right greater than left. Before and after each tDCS, single-pulse TMS and paired-pulse TMS were given to the left M1 and right M1 in order to assess the corticospinal excitability and mutual direction of IHI.

## Methods

### Participants

Participants were sixteen healthy right-handed volunteers (22–34 years old, 3 females). All participants gave their written informed consent to participate in this study. The experimental and consent procedures were approved by the ethical review board of the National Rehabilitation Center for Persons with Disabilities and which was in accordance with the guidelines established in the Declaration of Helsinki. All participants were naïve to the purpose of the experiments.

### Recordings

Electromyography (EMG) was recorded from the bilateral first dorsal interosseous (FDI) muscles. Self-adhesive Ag/AgCl electrodes were placed over the muscle belly and the metacarpophalangeal joint. The EMG signals were amplified and filtered (bandwidth, 20–3000 Hz) with a conventional bioamplifier (BIOTOP 6R12, NEC San-ei, Tokyo, Japan). Their digital data were acquired with a sampling rate of 5 kHz with a CED 1401 A/D converter (Cambridge Electronic Design, Cambridge, UK) and stored on a computer for off-line analysis.

### TMS

Corticospinal excitability and IHI were investigated by single-pulse and paired-pulse TMS, respectively. TMS was delivered to the left M1 and the right M1 with a figure 8-shaped coil (70-mm diameter) connected to a Magstim 200 (Magstim, Whitland, UK). The stimulus location was determined to be the hot spot where weak stimulation could elicit the largest motor evoked potential (MEP) in the FDI muscle. The coil was held tangentially over the scalp with the handle pointing backward and 45° lateral away from the midline. The resting motor threshold (RMT) was defined as the minimum stimulus intensity that produced MEPs that were greater than 50 µV in at least 5 out of 10 consecutive trials. For the single-pulse TMS, the intensity of test stimulation (TS) was set at 120% of the RMT. Stimuli were consecutively delivered about every 10 s. Both the left and right hemispheres were examined sequentially with a randomized order across the participants. Fifteen MEPs were obtained at each hemisphere. Paired-pulse TMS was used to elicit IHI both from the left M1 to the right M1 and from the right M1 to the left M1. A suprathreshold conditioning stimulation (CS) with an intensity at 120% of RMT was delivered to M1 on one side 10 ms before a TS was delivered to M1 on the other side. For a few participants, it was impossible to place both coils at the optimal direction due to the size of the coil. Thus, the handle of the coil for the CS was pointed backward and more than 45° away from the midline until both coils did not contact each other. The TS intensity was adjusted so that the peak-to-peak amplitude of the MEP was about 1 mV. The paired-pulse stimulation and TS alone were randomly given every 10 s. Fifteen control MEPs and 15 conditioned MEPs were obtained at each side of tested FDI. For both single-pulse and paired-pulse TMS, if trials showed more than 20 µV of EMG activity in the window of 100 ms before TMS, additional stimuli were given instead of those trials.

### tDCS

**Figure 1:**
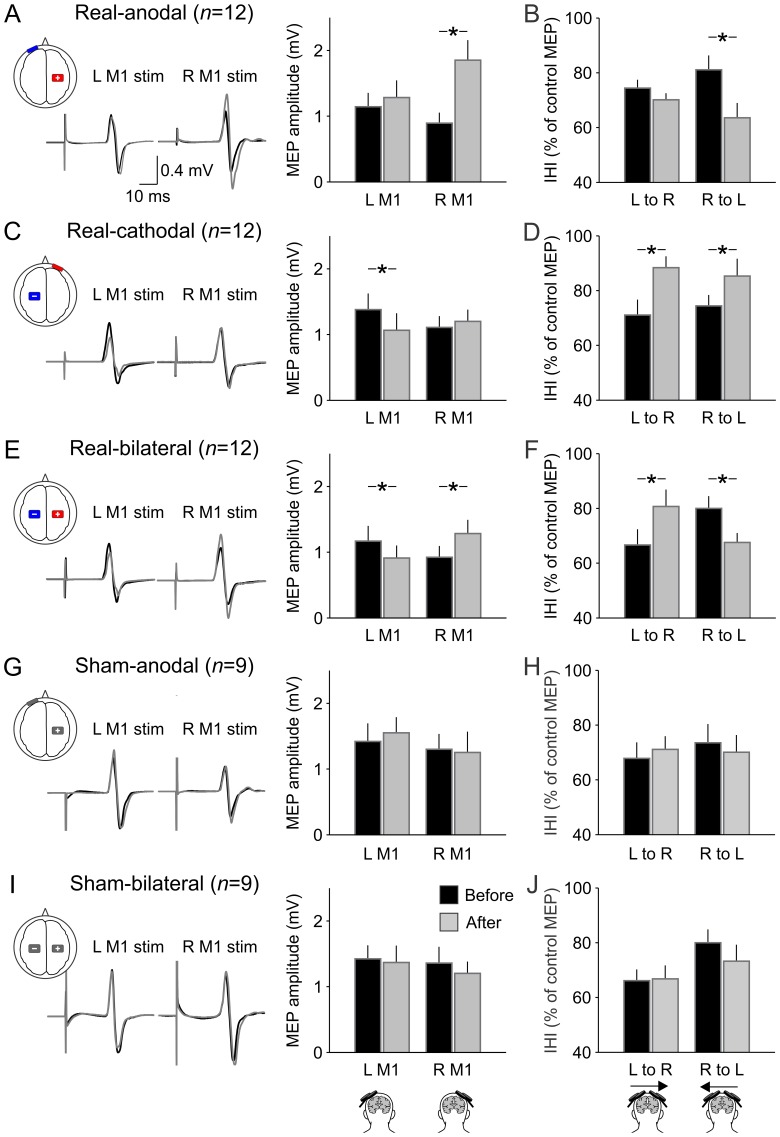
tDCS after-effects on MEPs and IHI. From top to bottom, real-anodal tDCS (A, B), real-cathodal tDCS (C, D), real-bilateral tDCS (E, F), sham-anodal tDCS (G, H), and sham-bilateral tDCS (I, J). The left and right sides of the traces are MEPs that are elicited by single-pulse TMS over the left M1 and right M1, respectively. The black and gray lines indicate MEPs that were elicited before and after DCS, respectively. The left bar graphs (A, C, E, G, J) show the average data of MEP of all participants. The sets of the left- and the right-sided columns represent MEP amplitude elicited by left (L) M1 stimulation and right (R) M1 stimulation, respectively. The rights bar graphs (B, D, F, H, J) show the average data of IHI of all participants. IHI was expressed as the ratio of the conditioned MEP amplitude normalized by the control MEP amplitude (i.e., larger value indicates less IHI). The sets of the left- and right-sided columns represent IHI from the left M1 to the right one (L to R) and that from the right M1 to the left one (R to L), respectively. The black and gray columns represent before and after tDCS, respectively. Error bas show standard error of means. The asterisks indicate a significant difference; * *p*<0.05.

Direct current stimulation was delivered by a battery-driven constant-current stimulator (Eldith DC-Stimulator, NeuroConn, Ilmenau, Germany) through a pair of rubber electrodes (5×5 cm) covered with saline-soaked sponges (5×6 cm). We examined three kinds of electrode montages; anodal tDCS over the right M1, cathodal tDCS over the left M1, and bilateral tDCS over the right M1 and left M1. For anodal tDCS, the anode and cathode were positioned on the right M1 (i.e., the hot spot of the left FDI) and the superior edge of the left orbit, respectively (Figure 1A). For cathodal tDCS the anode and cathode were positioned on the superior edge of the right orbit and the left M1 (i.e., the hot spot of the right FDI), respectively (Figure 1B). For bilateral tDCS, the anode and cathode were over the right M1 and left M1, respectively (Figure 1C). The current polarity at each electrode was masked to the participants. 1.5 mA of direct current stimulation was delivered for 15 min. The current was gradually increased and decreased during the first and last 10 s of the stimulation, respectively. Sham-tDCS was conducted for 15 min with the montages of anodal tDCS and bilateral tDCS (Figure 1D, E). The 1.5 mA of direct current stimulation was delivered for first 30 s subsequent to 10 s of current increment.

### Experimental procedures

The experiments were composed of real-tDCS and sham-tDCS sessions. 12 participants joined the real-tDCS session and 9 participants joined the sham-tDCS session. 5 out of 16 participants were involved in both sessions; three of them participated in the real-tDCS session first and two of them participated in the sham-tDCS session first. Each kind of electrode montage was tested on a different day. At least 3 weeks were interleaved across the experimental days. At each tDCS session, the order of the electrode montages was randomized across participants. In the experiments, the participants sat comfortably on a reclining chair with their shoulders and elbows semi-flexed. Both of their hands were placed on the table with palms downward. Before the tDCS application, RMT was measured in both M1. Then, the single-pulse and paired-pulse TMS protocols were conducted. After these baseline measurements were made, real- or sham-tDCS with an electrode montage was given for 15 min. After tDCS application, the same measurements were conducted on each side of M1.

### Data analysis

For the evaluation of corticospinal excitability, the peak-to-peak amplitudes of the MEPs elicited by single-pulse TMS were measured in the window 18–50 after the TMS trigger. The extent of after-effects was expressed as the ratio of the MEP amplitude obtained after tDCS to the baseline MEP amplitude obtained before tDCS. In order to evaluate IHI, the amplitude of the conditioned MEPs elicited by paired-pulse stimulation were normalized by the amplitude of the control MEPs evoked by TS alone. Trials with more than 20 µV of peak-to-peak amplitude in background EMG activity for 100 ms pre-stimulus period were discarded from the analysis. For the statistical analysis, a three-way analysis of variance (ANOVA) with repeated measures was performed with factors of time (before and after tDCS), tDCS type (real-anodal, real-cathodal, real-bilateral, sham-anodal, sham-bilateral), and TS side (left and right M1). In case a significant interaction between three factors was obtained, appropriate follow-up two-way ANOVA was conducted to examine the interaction of time and TS side factors at each tDCS type. In order to compare the magnitude of after-effects across conditions, one-way ANOVA with repeated measures was conducted with factor of tDCS type at each TS side. For the comparison of baseline level in each measurement, two-way ANOVA with repeated measures was performed with factors of tDCS type and TS side. *Post-hoc* comparisons were conducted by Tukey's test.

According to the findings in the previous studies [12], [16], we expected that real-tDCS induced the polarity-specific modulation in the M1 underneath the active electrode. Thus, we anticipated that real-tDCS influenced the excitability of callosal neurons in the same M1. Therefore, to examine the relationship between the after-effects on MEP amplitude and IHI, we also conducted Pearson correlation analysis in the real-tDCS after-effects between MEP amplitude and IHI. *P* values less than 0.05 were recognized as statistically significant in all analyses. Group data are presented as the mean ± standard deviation in the text.

## Results

### RMT, MEP

RMT was different across TS sides (*F*_1,49_ = 5.53, *p* = 0.02). TMS given to the left M1 showed slightly lower RMT than the right M1 (Table 1). However, tDCS did not affect RMT (*F*_1,49_ = 0.43, *p* = 0.51) regardless of tDCS type (*F*_4,49_ = 0.70, *p* = 0.59). Three-way ANOVA did not show any significant interactions (time × tDCS type, *F*_4,49_ = 0.07, *p* = 0.99; time × TS side, *F*_1,49_ = 0.26, *p* = 0.62; tDCS type × TS side, *F*_4,49_ = 0.24, *p* = 0.92; time × tDCS type × TS side, *F*_4,49_ = 1.00, *p* = 0.42).

**Table 1:** Resting motor threshold (% maximal stimulator output).

		Real-tDCS (*n* = 12)			Sham-tDCS (*n* = 9)	
		Anodal	Cathodal	Bilateral	Anodal	Bilateral
Left M1	Before	43.8±6.1	44.7±6.9	43.8±6.8	46.1±7.6	46.6±7.5
	After	43.5±5.5	45.0±7.8	44.4±6.5	46.0±7.6	46.0±7.6
Right M1	Before	44.7±6.0	46.8±9.8	45.7±8.1	48.3±7.6	48.4±5.6
	After	44.2±6.5	46.1±10.7	44.7±8.9	48.6±7.0	48.9±6.7

Values are mean ± standard deviation.

Figure 1A illustrates representative example of MEPs elicited before and after tDCS. Consistent with the findings in the previous studies [12], [16], facilitation and inhibition were observed in the MEPs elicited by single-pulse TMS over the M1 under the anode and the cathode, respectively. Three-way ANOVA revealed significant interactions of time and tDCS type and TS side (*F*_4,49_ = 4.39, *p* = 0.004) on MEP amplitude, indicating that the interaction of time and TS side was dependent on the tDCS type. Then, we performed follow-up two-way ANOVA for each tDCS type. Regardless of electrode montage, real-tDCS showed significant interaction of time and TS side (real-anodal, *F*_1,11_ = 8.32, *p* = 0.02; real-cathodal, *F*_1,11_ = 5.76, *p* = 0.04; real-bilateral, *F*_1,11_ = 23.53, *p*<0.001), indicating that all electrode montage had tDCS after-effect on MEP amplitude such that their tDCS after-effects were different depending on the TS side. *Post-hoc* analysis revealed that after real-anodal tDCS over the right M1, the MEP elicited from the right M1 was increased (232.0±144.7%, *p*<0.001) and the MEP elicited from the left M1 was unchanged (111.1±41.7%, *p* = 0.54) compared with the baseline (Figure 1A). After real-cathodal tDCS over the left M1, the MEP elicited from the left M1 was decreased (76.2±27.6%, *p* = 0.01) and the MEP elicited from the right M1 was unchanged (109.0±36.5%, *p* = 0.45, Figure 1C). After real-bilateral tDCS (anode over the right M1, cathode over the left M1), the MEP elicited from the right M1 was increased (157.6±68.2%, *p*<0.001) and the MEP elicited from the left M1 was decreased (75.4±28.3%, *p* = 0.01, Figure 1E). In contrast to real-tDCS, nether of sham-tDCS showed significant main effect of time (sham-anodal, *F*_1,8_ = 0.38, *p* = 0.55; sham-bilateral, *F*_1,8_ = 1.36, *p* = 0.28) or TS side (sham-anodal, *F*_1,8_ = 1.17, *p* = 0.31; sham-bilateral, *F*_1,8_ = 0.66, *p* = 0.44), or their interaction (sham-anodal, *F*_1,8_ = 2.68, *p* = 0.14; sham-bilateral, *F*_1,8_ = 0.05, *p* = 0.84, Figure 1G, I). Two-way ANOVA revealed that baseline level of MEP amplitude before tDCS was not different across tDCS types (*F*_4,49_ = 0.96, *p* = 0.44) or TS sides (*F*_1,49_ = 3.79, *p* = 0.06) with no interaction of their factors (*F*_4,49_ = 0.07, *p* = 0.99).

To sum up, facilitation and inhibition were observed in the MEPs elicited from the M1 under the anode and the cathode, respectively. With real-anodal and real-cathodal tDCS, the MEP elicited from the unstimulated M1 was unchanged. The magnitude of after-effects was not different across the conditions that showed significant facilitation (real-anodal 232.0±144.7%, real-bilateral 157.6±68.2%, *p* = 0.20) or inhibition (real-cathodal 76.2±27.6%, real-bilateral 75.4±28.3%, *p* = 0.99).

### IHI

Both before and after tDCS, IHI was examined both from the left M1 to the right M1 and from the right M1 to the left M1. By adjusting the TS intensity to elicit a 1 mV MEP, the amplitude of the control MEP was not different across conditions. Three-way ANOVA revealed significance of neither main effect of time (*F*_1,49_ = 0.07, *p* = 0.80), tDCS type (*F*_4,49_ = 0.1.74, *p* = 0.16), TS side (*F*_1,49_ = 0.33, *p* = 0.57), nor their interactions (time × tDCS type, *F*_4,49_ = 0.76, *p* = 0.59; time × TS side, *F*_1,49_ = 0.10, *p* = 0.76; tDCS type × TS side, *F*_4,49_ = 0.40, *p* = 0.81; time × tDCS type × TS side, *F*_4,49_ = 0.24, *p* = 0.91).

The three-way repeated measures ANOVA revealed significant interaction of time and tDCS type and TS side (*F*_4,49_ = 2.64, *p* = 0.04) on IHI, indicating that the interaction of time and TS side was dependent on the tDCS type. Then, we performed follow-up two-way ANOVA for each tDCS type. Real-anodal and real-bilateral tDCS showed significant interaction of time and TS side (real-anodal, *F*_1,11_ = 8.36, *p* = 0.02; real-bilateral, *F*_1,11_ = 20.08, *p*<0.001). On the other hand, real-cathodal tDCS had only main effect of time (*F*_1,8_ = 9.42, *p* = 0.01) but not main effect of TS side (*F*_1,8_ = 0.001, *p* = 0.98) or interaction of time and TS side (*F*_1,8_ = 1.78, *p* = 0.21). That is, in the real-tDCS session, all electrode montages had tDCS after-effect on IHI. The tDCS after-effect was different depending on the TS side (i.e., direction of IHI) after real-anodal and real-bilateral tDCS. On the other hand, the after-effect of real-cathodal tDCS was independent of TS side. *Post-hoc* analysis demonstrated that after real-anodal tDCS over the right M1, IHI from the right M1 to the left M1 was significantly increased compared with baseline (*p*<0.001). However, IHI from the left M1 to the right M1 was unchanged (*p* = 0.16, Figure 1B). After real-cathodal tDCS over the left M1, a reduction in IHI magnitude was observed both from the left M1 to the right M1 and from the right M1 to the left M1 (*p* = 0.01, Figure 1D). After real-bilateral tDCS (anode over the right M1, cathode over the left M1), IHI from the left M1 to the right M1 was decreased compared with baseline (*p* = 0.001). In contrast, IHI from the right M1 to the left M1 was increased compared with baseline (*p* = 0.003; Figure 1F). Again, neither of sham-tDCS affected IHI (Figure 1H, J). Two-way repeated measures ANOVA did not show any significant effect of time (sham-anodal, *F*_1,8_ = 0.0003, *p* = 0.99; sham-bilateral, *F*_1,8_ = 0.28, *p* = 0.61), TS side (sham-anodal, *F*_1,8_ = 0.11, *p* = 0.75; sham-bilateral, *F*_1,8_ = 5.10, *p* = 0.06), or their interaction (sham-anodal, *F*_1,8_ = 0.62, *p* = 0.45; sham-bilateral, *F*_1,8_ = 1.68, *p* = 0.23). Baseline level of IHI before tDCS was generally larger from the left M1 to the right M1 than the opposite direction. Two-way repeated measures ANOVA showed significant effect of TS side on the baseline level of IHI (*F*_1,49_ = 10.78, *p* = 0.002), but not main effect of tDCS type (*F*_4,49_ = 0.40, *p* = 0.81) or interaction of tDCS type and TS side (*F*_4,49_ = 0.67, *p* = 0.62), indicating that although an asymmetry of IHI was observed across IHI directions, the baselines of IHI on each direction was similar level across tDCS types.

In summary, IHI from the M1 under the anode was increased. In contrast, IHI from the M1 under the cathode was decreased. IHI from the unstimulated M1 showed a decrease after cathodal tDCS, but it was unchanged after anodal tDCS. Finally, we tested the correlation of tDCS after-effects between MEP amplitude and IHI. However, we did not find any significant correlations between the modulations of MEP amplitude and IHI regardless of TS side (Table 2).

**Table 2:** Relationships in the after-effects of real-tDCS on MEP amplitude and IHI.

Measurements		Electrode montage					
		Anodal		Cathodal		Bilateral	
MEP	IHI	*r* value	*p* value	*r* value	*p* value	*r* value	*p* value
Left M1	Left to Right M1	−0.21	0.52	0.35	0.27	0.16	0.62
Left M1	Right to Left M1	−0.01	0.98	−0.30	0.35	−0.28	0.39
Right M1	Left to Right M1	−0.36	0.25	0.05	0.89	−0.27	0.39
Right M1	Right to Left M1	0.21	0.52	0.03	0.92	−0.32	0.31

Values were obtained by Pearson correlation analysis.

## Discussion

The present study demonstrated that tDCS produced polarity-specific after-effects on IHI from the stimulated M1 at which the corticospinal excitability was changed. Regardless of unilateral or bilateral tDCS, IHI was generally increased from the M1 at which the corticospinal excitability was increased and decreased from the M1 at which the corticospinal excitability was decreased. Bilateral tDCS simultaneously produced the opposite directional modulation in IHI from the left to the right M1 and in IHI from the right to the left M1 in addition to the bidirectional corticospinal modulation. Although unilateral anodal tDCS did not affect the corticospinal excitability at the side of unstimulated hemisphere or IHI from the M1 on that unstimulated hemisphere, unilateral cathodal tDCS suppressed IHI from the M1 on the unstimulated hemisphere even though the corticospinal excitability was unchanged at the unstimulated side.

In most cases, the modulations of IHI were parallel to the modulations of corticospinal excitability at the side sending callosal volleys. Thus, it is likely that the tDCS after-effects on IHI are relevant with the excitability change in the motor cortex sending callosal volleys. However, we did not observe any significant relationships between the modulations of MEP amplitude and IHI. If IHI is mainly derived from the collateral discharges of corticospinal neurons and the tDCS-induced modulation in IHI resulted from the changes in collateral discharges, the modulations in MEP amplitude and IHI could have been correlated. Therefore, the modulation of transcallosal pathways could be partly independent of the changes in corticospinal descending pathways. Transcallosal inhibition is assumed to be derived from the discharge of callosal neurons that are distinct from corticospinal neurons [24], [28], [29]. Accordingly, tDCS might have similarly influenced both corticospinal and callosal neurons in the same M1. In some previous studies, IHI has been evaluated by matching the size of CS-induced MEPs in order to normalize the CS effect [24], [30], [31]. However, the adjusted CS intensity may not be sensitive enough to detect the excitability change in callosal neurons when both corticospinal and callosal neurons are modulated in parallel [24], [31], [32]. In the present study, we used the same CS intensity across before and after the tDCS sessions according to the RMT. Therefore, the modulation of IHI could be observed by detecting parallel modulation in the excitabilities of corticospinal and callosal neurons.

Our findings of the modulation of transcallosal inhibition are partly inconsistent with a previous study that used iSP [25], although the corticospinal excitability was modulated in a similar way. The previous study did not observe changes in iSP from the modulated M1 underneath the tDCS electrode [25]. One possible explanation for this discrepancy may be the differences in the tDCS parameters. The present experiments used a higher intensity (1.5 mA) and a longer duration (15 min) of tDCS compared to the previous study (1.0 mA intensity, 10 min duration). The tDCS after-effects have been shown to increase up to a certain extent of intensity and duration [12], [33], [34]. Furthermore, because the threshold for eliciting transcallosal inhibition is known to be higher than the RMT for MEPs [29], [35]–[37], callosal neurons might require a relatively high intensity and long duration of tDCS to be modulated. Another possibility is the different neural populations mediating transcallosal inhibition because partly different sets of callosal neurons and target neurons receiving callosal volleys have been assumed to mediate short-interval IHI and iSP [26]. In addition, iSP appears as the inhibition of static voluntary activity, although IHI is the inhibition of synchronized corticospinal discharges that TMS artificially evokes [29]. Accordingly, such physiological differences might relate to the different susceptibilities to tDCS. Indeed, previous study using rTMS demonstrated the modulation of IHI without robust changes of iSP [27].

We also observed a reduction of IHI from the unchanged M1 after unilateral cathodal tDCS, although unilateral anodal tDCS did not modulate IHI from the unchanged M1. These findings suggested that unilateral tDCS affected interneuronal circuits that presynaptically regulate callosal transmission and/or relay them to the corticospinal neurons [25]. Indeed, tDCS-induced plastic modulation has been shown in some intracortical interneurons that mediate gamma-aminobutyric acid activity [38]–[40]. One potential reason that unilateral anodal tDCS failed to modulate IHI in this direction might be due to the asymmetry in transcallosal inhibition. Generally, transcallosal inhibition is greater from the left M1 to the right M1 than from the right M1 to the left M1 in right-handers [41], [42], which was also confirmed in our study. Furthermore, previous study reported asymmetric effects of tDCS [4]; tDCS applied over the left dominant hemisphere was more effective than that over the right non-dominant hemisphere. In our study, anodal and cathodal stimuli were given to the different hemispheres. Hence, the lack of modulation of IHI toward the facilitated right-side M1 might be also attributed to the decreased efficiency of tDCS that is applied over the non-dominant hemisphere.

The effect of interventional brain stimulation on transcallosal inhibition has been tested by several stimulation protocols such as low-frequency rTMS [27], [43], theta burst stimulation [44], [45], paired associative stimulation [46], tDCS [5], [25], and quadripulse TMS [47]. Even though their protocols were able to elicit bidirectional modulation on the corticospinal excitability, the modulation of transcallosal inhibition was not always observed [44], [45]. Presumably, the neural elements involving with transcallosal inhibition might have different susceptibilities according to the type of brain stimulation protocol. Although our results show that bilateral tDCS was able to elicit the bidirectional modulation in transcallosal inhibition between left M1 and right M1 in addition to the left and right corticospinal excitabilities, it is worth noting that the extent of MEP modulation by bilateral tDCS was not different compared to that by unilateral tDCS. This finding was also reported in recent studies [16], [17]. Additionally, in line with previous studies [16], [25], [48], neither the polarity of unilateral tDCS affected the corticospinal excitability in the contralateral unstimulated motor cortex even though transcallosal inhibition toward that motor cortex showed short-lasting after-effects (Figure 1). These findings suggest that transcallosal inhibition modulated by tDCS might have minor static effects on the corticospinal excitability in the contralateral motor cortex. Nevertheless, previous studies demonstrated that bilateral tDCS was more effective for improving hand motor performance compared to unilateral anodal tDCS over the target motor cortex [3], [10], and that unilateral cathodal tDCS over a motor cortex results in substantial improvement of ipsilateral hand motor function in healthy [2], [4] and stroke individuals [7], [9], [11], [49]. These facts could provide us rationale to suppose that suppressed transcallosal inhibition contributes to the contralateral cortical motor activity. Indeed, Williams et al. [5] demonstrated a functional relationship between the suppression of transcallosal inhibition and improvements in motor performance using bilateral tDCS. Conceivably, it might be that a functional role of the decreased transcallosal inhibition can be observed in a time-specific motor event like movement initiation. Transcallosal inhibition is gradually decreased according to the time course of movement initiation [50], [51]. Therefore, a sustained reduction of transcallosal inhibition could contribute to such a situation of motor performance rather than a static enhancement of corticospinal excitability. To support this notion, recent studies using functional magnetic resonance imaging demonstrated that motor task-related M1 activation was greater in bilateral tDCS compared to unilateral anodal tDCS, and that the M1 activation changes in laterality were correlated with microstructural status of transcallosal motor fibers [18] although resting-state interhemispheric functional connectivity between the left M1 and the right M1 did not show after-effects regardless of unilateral anodal or bilateral tDCS [20]. Therefore, it seems conceivable that modulated transcallosal pathways contribute to the motor performances without marked changes in the corticospinal excitability at rest.

From the methodological point of view, we need to consider tDCS parameters as limitations of our study. First, strong intensity and long duration of direct current stimulation has a risk of over stimulating that causes reversing facilitatory effect of cathodal tDCS on the corticospinal excitability. A recent study demonstrated that cathodal tDCS with 2 mA of intensity and 20 min of duration facilitated the corticospinal excitability [52]. Because tDCS with a high intensity (2 mA) and a short duration (5 min) retained the general polarity-specific after-effects [16], the combination of intensity and duration might be a specific factor for the tDCS after-effects. Second, small number of participants should be considered as another limitation. Though we found significant tDCS after-effects on MEP amplitude and IHI, some insignificant results may be due to small sample size. We should make a point that the participants were not completely identical across real-tDCS and sham-tDCS sessions. Finally, our study cannot completely rule out spinal effects [53], [54]. Though IHI was demonstrated to be mediated by cortical circuits through transcallosal pathways [55], [56], potential contribution of subcortical circuits to IHI need to be considered [57].

As a therapeutic tool, tDCS has been frequently applied in patients with hemiparetic stroke [58]. Thus, our findings that tDCS modulated transcallosal inhibition with polarity-specific manner could provide a useful perspective on the understanding of the tDCS therapeutic effect on the recovery of motor function after stroke. In terms of interhemispheric neural modulations, the application of cathodal tDCS to contralesional hemisphere appears to be reliable as demonstrated by some clinical studies [7], [8], [10], [11], [49]. However, we may also need to take into account the tDCS effect on the uncrossed ipsilateral motor pathway [59], [60]. A recent study demonstrated that cathodal tDCS over a motor cortex affected presumed uncrossed cortico-propriospinal pathway [60]. As the severely impaired motor function is potentially compensated by ipsilateral cortical activity [61], it is important to note the potential risk that cathodal stimulation over ipsilesional hemisphere deteriorates motor function [62].

In conclusion, the present study demonstrated that tDCS produced polarity-specific after-effects on transcallosal inhibition between motor cortices. Comprehensively, IHI was increased from the M1 at which the corticospinal excitability was increased and decreased from the M1 at which the corticospinal excitability was decreased, suggest that tDCS is capable of modulating neuronal activities that are involved with sending and receiving callosal discharges.
